# IRF4 as a novel target involved in malignant transformation of oral submucous fibrosis into oral squamous cell carcinoma

**DOI:** 10.1038/s41598-023-29936-8

**Published:** 2023-02-16

**Authors:** Li Meng, Yucheng Jiang, Jiawen You, Panpan Zhao, Weiguang Liu, Na Zhao, Zhichun Yu, Junqing Ma

**Affiliations:** 1 Jiangsu Key Laboratory of Oral Diseases, Nanjing Medical University, 140 Hanzhong Road, Nanjing, 210029 China; 2 Department of Biochemistry, School of Life Sciences, Nanjing Normal University, Nanjing, 210023 China; 3 Green Hope High School, Cary, NC 27519 USA; 4 Department of Orthodontics, Affiliated Hospital of Stomatology, Nanjing Medical University, Nanjing, 210029 China

**Keywords:** Cancer, Immunology, Biomarkers, Oncology, Pathogenesis

## Abstract

Oral squamous cell carcinoma (OSCC) in the context of oral submucous fibrosis (OSF) has a high incidence owing to undefined pathogenesis. Identifying key genes and exploring the underlying molecular mechanisms involved in the conversion of OSF into OSCC are in urgent need. Differentially expressed genes (DEGs) between OSCC and OSF were dug from GEO databases and a total of 170 DEGs were acquired. Functional association of DEGs were analyzed by GO and KEGG. Protein–protein interactions (PPIs) analysis was carried out and candidate biomarkers were identified by Gene co-expression analysis and Cox analyses. Hub genes were confirmed by qRT-PCR in tissues and cell lines, of which we found that IRF4 mRNA was successively up-regulated from Normal to OSF and then to OSCC and associated with immune infiltrating levels. In addition, Immunohistochemical (IHC) and Immunofluorescence (IF) assays were conducted to validate the consistent upregulation of IRF4 and the oncogene role of IRF4 in OSF and OSCC at translation level. IRF4 may be indicative biomarker in transformation of OSF into OSCC. High IRF4 expression contribute to increased immune infiltration of OSCC and may provide a novel diagnostic marker for OSCC patients translated from OSF.

## Introduction

It is estimated that 10–20% of the world's population is accustomed to chewing betel nut. Epidemiological study results suggested that betel nut is the main cause of oral submucosal fibrosis (OSF), and consumption of betel nut is also associated with oral squamous cell carcinoma (OSCC)^1,2^. OSF is a precancerous condition with a propensity for malignant transformation and up to a quarter of cases are present with epithelial dysplasia at biopsy. Malignant transformation rates have been estimated to range from 5.6 to 9.13 percent in recent studies^3,4^. OSCC is the most common malignant tumor of the head and neck^5^, and also the sixth highest incidence of cancer worldwide^6,7^. It is further observed that OSCC originating from OSF tends to occur in young adults, commonly in the posterior buccal, gingival and vestibular mucosa, and are more clinically aggressive and metastatic^8^. The prognosis and clinicopathological features of OSCC patients with OSF are inferior to conventional OSCC patients^9^, so it is urgent to search for new OSCC diagnostic biomarkers in the context of OSF.

The role of tumor immunology in tumorigenesis and progression is significant. Numerous types of cancer form ectopic lymphoid aggregates, also called tertiary lymphoid structures (TLSs), which are relevant to superior prognosis and response to immunotherapy^10^. The feature of tumor microenvironment (TME) is nutrition competition or coordination between tumor and infiltrating immune cells that influences antitumor immunity. Adaptive immune responses are essential for the clearance of tumors^11^. In the aspect of OSCC, inflammatory mediators are identified as potential markers for diagnosis and prognosis of OSCC^12^. The tumor microenvironment impacts evasion of OSCC from immune recognition and destruction^13^.

As a member of the IRF transcription factor family, interferon regulatory factor 4 (IRF4) is expressed and crucial for the development and function of numerous immunocyte types such as B cell, T cell and dendritic cell. IRF4 plays a significant role in autoimmune diseases^14^. In various mature lymphoid neoplasms, abnormally expressed IRF4 also acts as an oncogene. IRF4 and its upstream factor NF-κB form a regulatory circuit to promote the oncogenic transcriptional program in malignant lymphoid cells^15^. However, research on the role of IRF4 in OSCC is still lacking^16^.

In this study, we conducted a comprehensive bioinformatics analysis including functional enrichment analysis, CNV and immune infiltration analysis through Gene Expression Omnibus (GEO) and The Cancer Genome Atlas (TCGA) databases to identify key genes and explore the underlying molecular mechanisms involved in OSF into OSCC. We finally found the role of IRF4 in predictabilities of the transformation from OSF to the presence of OSCC. Understanding of the potential oncogenic axis may enable the discovery of noninvasive disease-specific diagnostic biomarkers. There is a discovery that IRF4 can be employed as a possible diagnostic and immunological predictor of malignant transformation of OSF into OSCC. This study may broaden the application of IRF4 in immunotherapy.

## Materials and methods

### Cell culture

Two human cell lines (HOK, HN4) were obtained from ATCC. The HN4 (OSCC cell line) and human oral keratinocytes (HOK, Normal control) cell line were cultured in Dulbecco’s modified Eagle’s medium containing 10% fetal bovine serum (Gibco, C11995500BT) and 1% penicillin/streptomycin (NCM Biotech, C125C5). Cells were incubated at 37 °C with 5% CO_2_.

### Clinical samples

This study was approved by the ethical committee department of Affiliated Hospital of Stomatology of Nanjing Medical University (PJ2022-086-001). Research was performed in accordance with relevant guidelines and regulations. Informed consent was obtained from all patients. A total of 8 Normal oral mucosa tissues (Normal) and 10 oral squamous cell carcinoma (OSCC) samples were obtained in Stomatological College of Nanjing Medical University. The normal oral mucosa tissues were collected from the wounds of patients from whom impacted third molar were extracted. 10 oral submucosal fibrosis (OSF) samples were collected at Hunan Xiangya Hospital of Central South University (202005151). Written informed consent was acquired from all of the patients.

### Gene expression profile of OSF and OSCC

Two datasets of gene expression profiles (GSE64216, GSE23558) were obtained from the Gene Expression Omnibus (GEO) database (http://www.ncbi.nlm.nih.gov/geo/)^17–19^. GSE64216 was downloaded from GPL10558 (Illumina HumanHT-12 V4.0 expression beadchip), which included 4 tissues from patients with oral submucous fibrosis and 2 from healthy controls. GSE23558 was downloaded from GPL6480 (Agilent-014850 Whole Human Genome Microarray 4 × 44 K G4112F), which contained 27 OSCC tissues compared with 4 independent controls and 1 pooled control oral cavity tissue from healthy donors.

Besides, the data of exon RNAseq and the corresponding clinical data of patients with OSCC were publicly available at TCGA (https://portal.gdc.cancer.gov/). A total of 505 OSCC patients’ tissues and 24 Normal tissues were included from TCGA-OSCC project.

### Screening of differentially expressed genes

In the GEO database, the GEO2R online analysis tool based on GEO query and R software (version R 4.2.0, https://www.r-project.org/) Limma package was used to analyze DEGs in the two comparisons (OSF vs. Normal, OSCC vs. Normal)^20^. The adj. P value was obtained through multiple checking and correcting by the Benjamini and Hochberg method^21^. DEGs in the two comparisons with the filter criteria of adj. *P* value < 0.05 and |logFC (fold change)|> 1 may be candidates in the development of OSF and OSCC. Likewise, differentially expressed mRNAs between Normal and tumor in the TCGA database were also obtained.

### Functional annotation of differentially expressed genes

Gene Ontology (GO) annotation^22^ and Kyoto Encyclopedia of Genes and Genomae (KEGG) pathway^23–25^ were used for the database for annotation, visualization and integrated discovery (https://www.bioinformatics.com.cn). For DEGs, *P* value < 0.05 and enrichment count of at least 5 were set as the cutoff criteria for significance during the development of OSF to OSCC.

### Protein–protein interaction (PPI) network analysis

Protein–protein interaction (PPI) networks were mapped to predict the interactions between gene–encoded proteins using the Search Tool for the Retrieval of Interacting Genes database (STRING, Version: 11.5) database^26^. A threshold of 0.4 (medium confidence) was used to construct the PPI network with Cytoscape software (version 3.7.2, http://chianti.ucsd.edu/cytoscape-3.7.2/)^27^. The degree of connectivity was employed to describe the association between nodes.

### Gene set enrichment analysis

In order to explore the biological signaling pathway, we ranked gene difference and performed gene set enrichment analysis based on their rankings. We used clusterProfiler and GSEABase to analyze the enriched pathways^28,29^. For the core candidate genes, we ranked the most overlapped enriched GO and KEGG pathways and visualized them. We used ggplot2 package in R to visualize the top-ranked enriched pathways^30^.

### Mutation analysis

Univariate and multivariate Cox analysis and forest plot were conducted with Sangerbox (http://www.sangerbox.com/). Copy number variation (CNV) is the most frequent type of genetic variation occurring in cancers and associated with the occurrence and progression of tumors. The frequencies of copy number gains or losses were conducted and waterfall plot of the mutational landscape was generated using the Sangerbox. The plot was conducted with R.

### Survival and tumor filtrating immune cells analysis

To conduct survival analysis, clinical data and RNA expression data from TCGA dataset were downloaded. Then Kaplan–Meier survival plot and LogRank analysis was done by GEPIA2 database (http://gepia2.cancer-pku.cn/#index)^31^. The correlation of IRF4 expression with tumor infiltrating cells were conducted with TIMER (https://cistrome.shinyapps.io/timer/) and TISIDB (http://cis.hku.hk/TISIDB/)^32,33^.

### RNA extraction and quantitative RT-PCR

Total RNA was extracted from HOK and HN4 cell line, OSCC and Normal oral mucosa tissues respectively using FastPure Cell/Tissue Total RNA Isolation Kit (Vazyme, RC112) according to the manufacturer’s protocols. Quantitative real-time PCR primers were designed and synthesized by Generay (Shanghai, China). The total RNA was converted into cDNA by Hiscript1 III RT SuperMix for qPCR (+gDNA wiper) (Vazyme, R312-01). The relative mRNA expression level and the internal control GAPDH were quantified on ABI PrismR 7900HT Real-Time PCR System (Applied Biosystems). All experiments were conducted three times independently, and the data were analyzed by the 2^−△△Ct^ method. The primer sequences used in qRT-PCR are shown in Table 1.

**Table 1:** Primer sequences used in qRT-PCR.

CFTR-qF	TGCCCTTCGGCGATGTTTTT
CFTR-qR	GTTATCCGGGTCATAGGAAGCTA
CXCL12-qF	ATTCTCAACACTCCAAACTGTGC
CXCL12-qR	ACTTTAGCTTCGGGTCAATGC
IRF4-qF	GCTGATCGACCAGATCGACAG
IRF4-qR	CGGTTGTAGTCCTGCTTGC
CD79A-qF	CAAGAACCGAATCATCACAGCC
CD79A-qR	TCTGCCATCGTTTCCTGAACA
PDGFA-qF	GCAAGACCAGGACGGTCATTT
PDGFA-qR	GGCACTTGACACTGCTCGT
FSTL3-qF	GTGCCTCCGGCAACATTGA
FSTL3-qR	GCACGAATCTTTGCAGGGA
LAMC2-qF	CAAAGGTTCTCTTAGTGCTCGAT
LAMC2-qR	CACTTGGAGTCTAGCAGTCTCT
PLAUR-qF	TGTAAGACCAACGGGGATTGC
PLAUR-qR	AGCCAGTCCGATAGCTCAGG
SEMA3C-qF	TTTGCGTGTTGGTTGGAGTAT
SEMA3C-qR	TCCTGTAGTCTAAAGGATGGTGG
TNFRSF12A-qF	TGCTTTGGCCCATCCTTGG
TNFRSF12A-qR	CTCCTGCGGCATCGTCTCC
CYP4F12-qF	GCATCCTGGCTTGGACCTATG
CYP4F12-qR	ACATCTGGGTCGAGTTCTTCA
FUT6-qF	GCCGACCGCAAGGTGTATC
FUT6-qR	CAGCCGTAGGGCGTGAAGA
HCG22-qF	CTCTTGGGACTCAGACAGGG
HCG22-qR	TGGGTGCTTAGTGGAAATG
HLF-qF	CCCTCGGTCATGGACCTCA
HLF-qR	ACTTGGTGTATTGCGGTTTGC
PTGDS-qF	GGCGTTGTCCATGTGCAAG
PTGDS-qR	GGACTCCGGTAGCTGTAGGA

### Immunohistochemical (IHC) staining

IHC was performed on formalin-fixed, paraffin-embedded specimens to quantify the levels of proteins encoded by the signature genes in Normal oral mucosa, OSF and OSCC tissues. The tissue sections were first deparaffinized in xylene and then dehydrated in ethanol. Citrate buffer (pH = 6) was used for antigen extraction, followed by blocking with goat serum for 1 h to prevent nonspecific binding of antibodies. The samples were then incubated in solutions containing specific primary antibodies (IRF4, 1:100, Abbkine) followed by incubation in solutions containing secondary antibodies. Finally, the sections were visualized after staining with 3, 3′-diaminobenzidine (DAB).

### Immunofluorescence (IF) staining

Tissue sections were dewaxed in xylene, and then dehydrated in ethanol. The tissues were then incubated in solutions containing specific primary antibodies (IRF4, 1: 100, abbkine) followed by incubation in 488 goat anti-rabbit secondary antibodies (1: 50, beyotime).

### Statistical analysis

The experimental results were presented as mean ± standard deviation (SD) and statistically analyzed using GraphPad Prism 9. The samples from the TCGA database were stratified into high- or low- expression groups according to the median of gene expression. Survival curves with log-rank test and Kaplan–Meier method were drawn for mRNAs. A threshold of *P* < 0.05 was determined statistically significant.

## Results

### Identification of DEGs in tissue type

**Figure 1:**
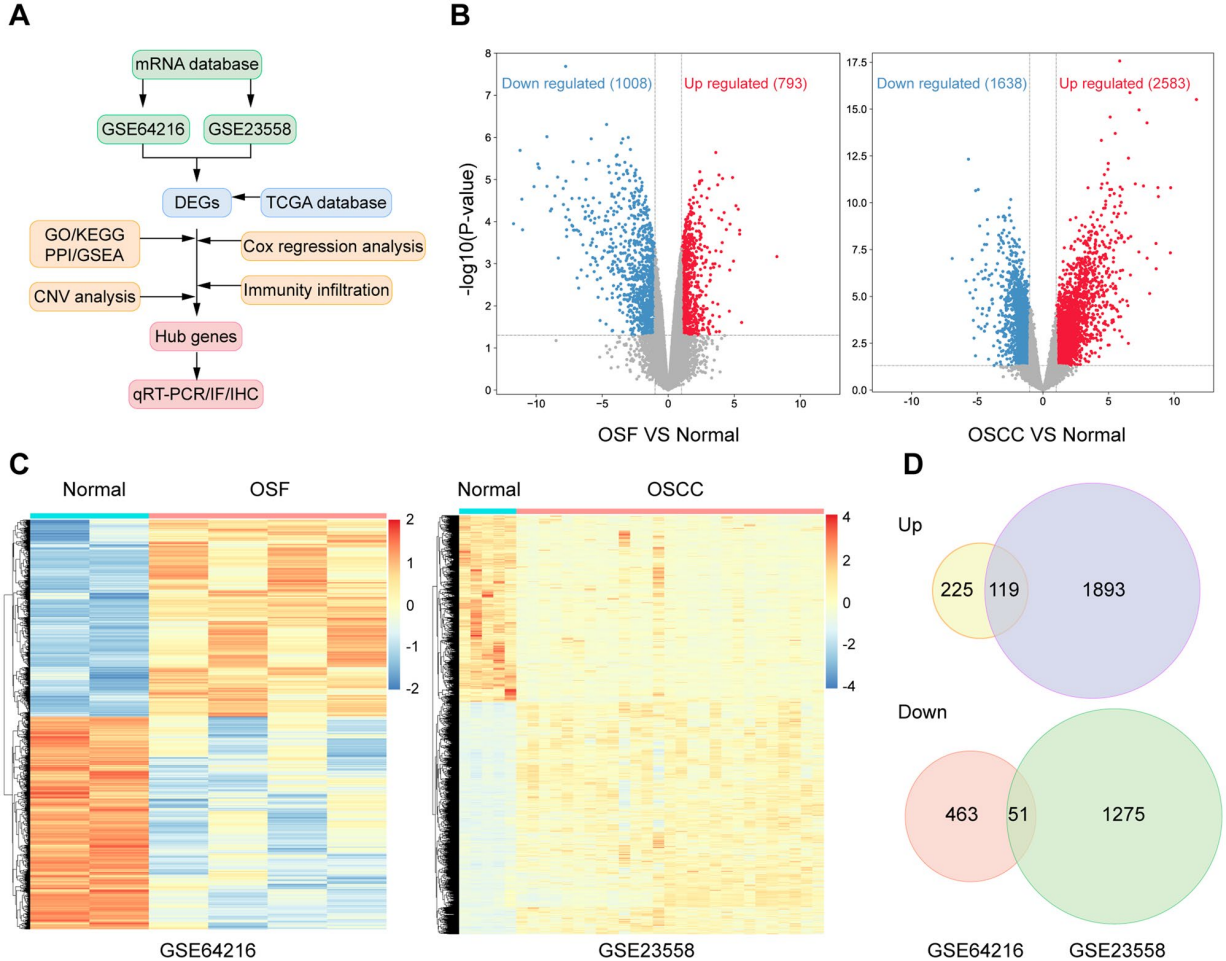
Identifcation of DEGs in GEO database. (**A**) Flow chart for identification of the hub genes connected to the transformation of oral submucous fibrosis (OSF) into oral squamous cell carcinoma (OSCC). DEGs, differentially expressed genes; GSE64216, including four oral submucous fibrosis patients and two controls; GSE42589, containing 27 OSCC samples and five control samples; TCGA, The Cancer Genome Atlas database; GO, gene ontology analysis; KEGG, Kyoto Encyclopedia of Genes and Genomes analysis; PPI, protein–protein interaction analysis; GSEA, Gene Set Enrichment Analysis; CNV, copy number variations; IF, immunofluorescence; IHC, immunohistochemistry. (**B**) The differentially expressed genes between Normal and OSF groups, Normal and OSCC groups in GSE64216 and GSE23558 analyzed by Volcano plots. Red, blue and gray color indicates high, low, and equal expression of mRNAs, respectively. (**C**) Heatmaps of differentially expressed genes in the GSE64216 and GSE23558. Red, high expression; blue, low expression. (**D**) The Venn diagram analysis of both down and both up-regulated DEGs in GSE64216 and GSE23558.

The processes used for screening OSCC and OSF-associated genes are shown in flow chart (Fig. 1A). Two microarray datasets, including GSE64216 (OSF vs. Normal) and GSE23558 (OSCC vs. Normal), were used to identify DEGs. A total of 858 DEGs were obtained and included 344 upregulated and 514 downregulated genes between Normal and OSF samples, and 3338 DEGs were acquired and contained 2012 upregulated and 1326 downregulated genes between Normal and OSCC samples (|logFC|> 2.0 and adj. *p* value < 0.05) (Fig. 1B, C). By integrating DEGs in two comparisons, 170 DEGs were overlapped in the two datasets including 119 upregulated and 51 downregulated genes, as shown in the Venn map (Fig. 1D).

### Functional correlation analysis and PPI analysis of DEGs

**Figure 2:**
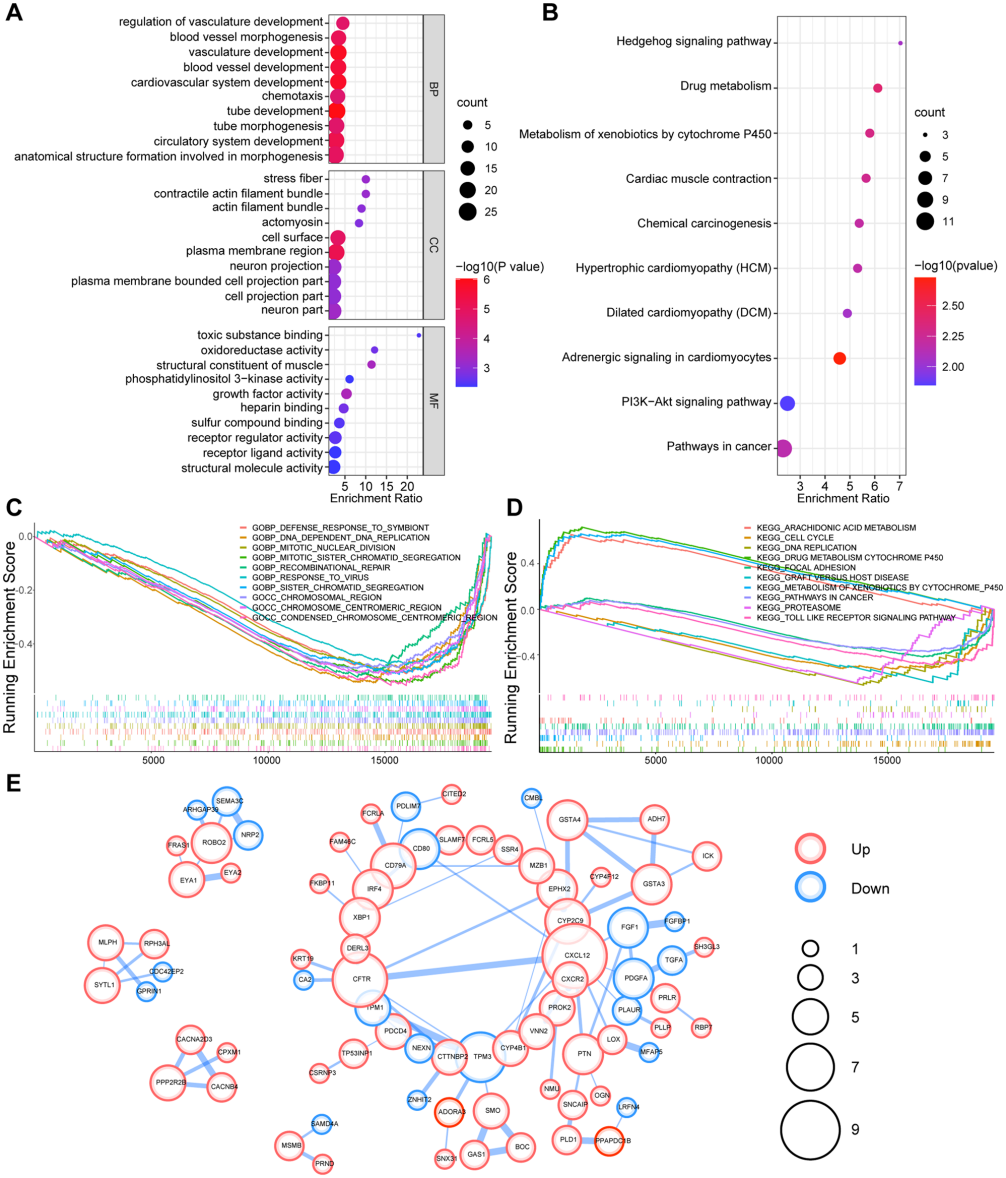
Functional correlation analysis and PPI analysis of DEGs. (**A**) Gene ontology (GO) enrichment analysis of overlapping DEGs. (**B**) KEGG pathways analysis of DEGs. Size of the dots represent Gene Counts, color of the dots are defined by the *p*-adjusted value. (**C**, **D**) The top 10 GO terms and KEGG pathways of GSE64216 and GSE23558 from GSEA analysis. (**E**) The PPI network of DEGs using the STRING database. Up- and down-regulated genes were marked in red and blue respectively.

GO enrichment and KEGG pathway analysis were performed to explore the overall effects of 170 DEGs in OSF and OSCC progression (|logFC|> 1 and FDR < 0.05). The GO annotations of DEGs consisted of three categories including biological process (BP), cellular component (CC), and molecular function (MF), which were used to analyze the functional enrichment of DEGs. The significant GO terms were conspicuously enriched in tube development, vasculature development, cardiovascular system development, blood vessel development, cell surface, plasma membrane region, stress fiber, growth factor activity, structural constituent of muscle, and heparin binding (Fig. 2A). The KEGG pathways were mainly enriched in adrenergic signaling in cardiomyocytes, drug metabolism, metabolism of xenobiotics by cytochrome P450, chemical carcinogenesis, etc*.* (Fig. 2B).

In addition, Gene set enrichment analysis (GSEA) analysis was conducted using GSEA software, which showed that “DEFENSE RESPONSE TO SYMBIONT, DNA DEPENDENT DNA REPLICATION, MITOTIC NUCLEAR DIVISION, etc*.*” GO terms (Fig. 2C) and “ARACHIDONIC ACID METABOLISM, CELL CYCLE, DNA REPLICATION, etc*.*” KEGG pathways were significant in OSCC compared with OSF and Normal samples (Fig. 2D). GSEA analysis of 858 DEGs in GSE64216 and 3338 DEGs in GSE23558 datasets were displayed in supplementary Fig. S1A and S1B respectively.

Co-expression analysis of these 170 DEGs were performed via construction PPI network, and the results was visualized through cytoscape. 5 protein coding gene clusters were obtained, of which the cluster centered around CFTR and CXCL12 may play a critical role in transformation of OSF into OSCC (Fig. 2E).

### Screening of hub genes assisted with TCGA database

**Figure 3:**
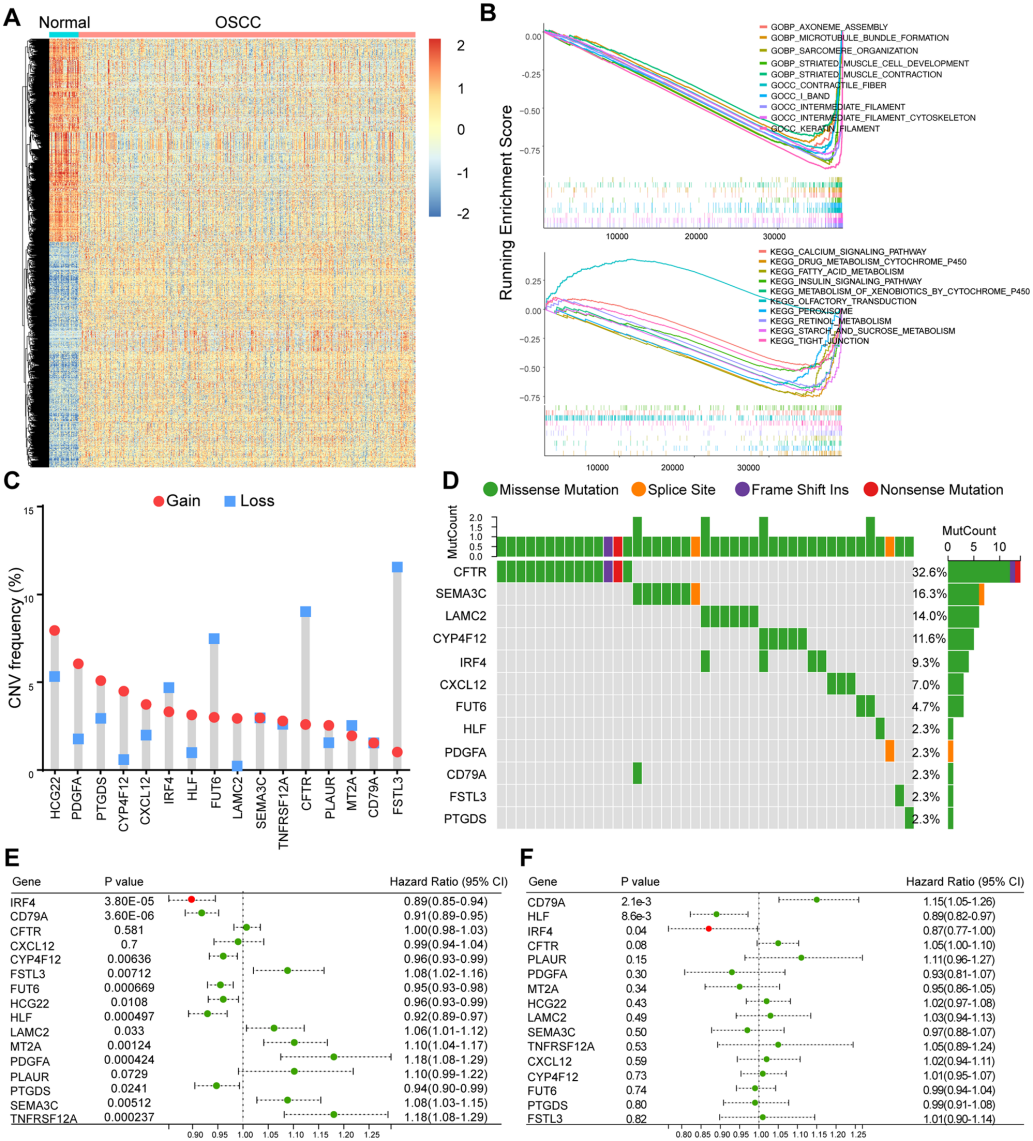
Screening of hub genes assisted with TCGA database. (**A**) Hierarchical clustering analysis of the differentially expressed genes between normal and OSCC from TCGA database. (**B**) GSEA analysis of OSCC in TCGA database. (**C**) Copy number variation (CNV) frequency of the 16 genes in TCGA cohort. CNV gain, red; CNV loss, blue color. The height of the column represents the alteration frequency. (**D**) Mutation frequency and classification of the 16 genes in OSCC. (**E**) Univariate Cox analysis of 16 DEGs with prognostic significance in TCGA OSCC datasets. (**F**) Multivariate Cox analysis 16 DEGs with prognostic significance in TCGA OSCC datasets.

The Cancer Genome Atlas (TCGA) OSCC cohort including 505 OSCC tissues and 24 Normal tissues was used to further screen hub genes (Fig. 3A). GSEA analysis displayed enriched GO terms and KEGG pathways in TCGA data (Fig. 3B, Fig. S1C). GEPIA database was adopted to detect the differential expression and prognostic value of DEGs (Figs. S2A, S3A). In a gene dependent network, the penetrance of a gene represents the number of genes associated with the phenotypic changes it affects. Therefore, genes with higher penetrance are more critical for phenotypic changes. In this work, we selected 16 candidate hub genes (CFTR, CXCL12, IRF4, CD79A, PDGFA, FSTL3, LAMC2, MT2A, PLAUR, SEMA3C, TNFRSF12A, CYP4F12, FUT6, HCG22, HLF, and PTGDS) as the central genes of the network for further analysis because of their high degree of connectivity (*P* < 0.05).

Copy number variation (CNV) is one of the main factors affecting gene expression abundance in many cancers^34–36^. Thus, we performed CNV frequency analysis for those 16 hub genes in TCGA data. Thus, we analyzed the incidence of CNV and somatic mutations of these hub genes in patients with OSCC in TCGA database, which revealed frequent CNV alterations in these genes, with a higher proportion of copy number losses than gains (Fig. 3C). The CNV mutation frequency of FSTL3 reached 10%, mainly with CNV deletion. This was followed by CFTR, FUT6, HCG22, IRF4, etc*.*, while the CNV mutation frequency in PDGFA, CYP4F12, CXCL12, HLF, LAMC2, PLAUR and CD79A was less than 2.5% (Fig. 3C). In addition, among the 16 hub genes, most of gene mutation were missense mutations. In detail, CFTR accounted for 32.6%, SEMA3C accounted for 16.3%, LAMC2 accounted for 14.0%, CYP4F12 accounted for 11.6 and IRF4 accounted for 9.3% (Fig. 3D).

To further explore whether 16 hub genes were associated with OSCC patient prognosis, we performed univariate and multivariate Cox regression analysis. The results of univariate Cox regression analysis revealed that 13 out of 16 genes were significant risk factors for overall survival (OS) of OSCC patients (*P* < 0.05) (Fig. 3E). Then, we conducted multivariate Cox regression analysis and selected three genes (*P* < 0.05) consisting of CD79A, HLF and IRF4 (Fig. 3F). Genes including HLF and IRF4 showed negative coefficients in the multivariate Cox regression analysis, implying low-risk signatures while CD79A showed the opposite effect. Taken together, IRF4 might be used as diagnostic biomarker for transformation of OSF into OSCC.

### Identification and validation of hub genes

**Figure 4:**
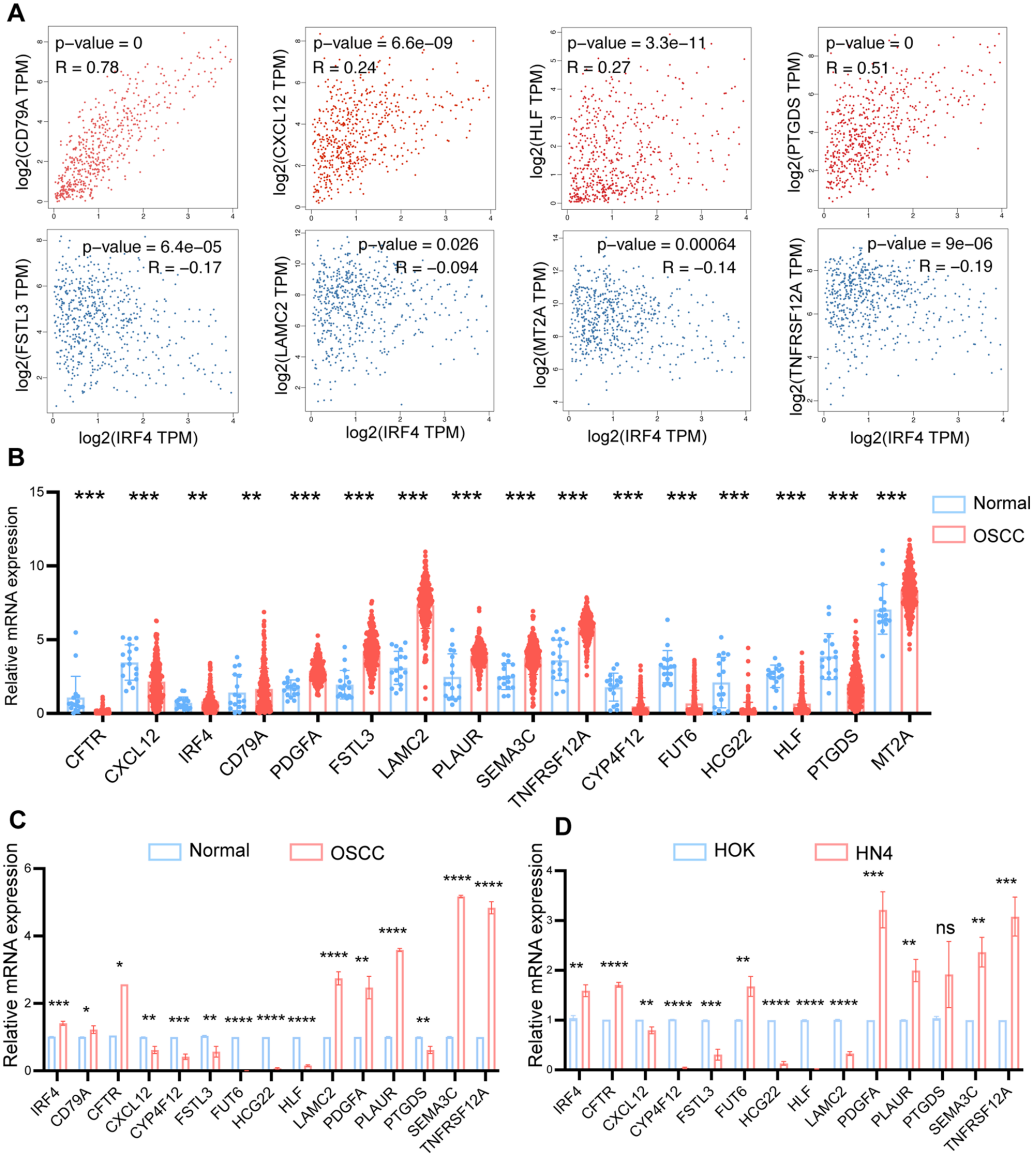
Identification and Validation of Hub Genes. (**A**) Correlation of IRF4 expression with other representative DEGs. (**B**) Expression data of DEGs in OSCC from TCGA database. (**C**) The mRNA expressions of DEGs by qRT-PCR in total RNA extracted from Normal and OSCC samples. (**D**) The mRNA expressions of DEGs by qRT-PCR in total RNA extracted from HOK and HN4 cell lines. ns, No significant; *p < 0.05; **p < 0.01; ***p < 0.001; ****p < 0.0001.

We calculated the levels of correlation between IRF4 and other 15 hub genes using GEPIA2 dataset and identified significant correlations between IRF4 and 8 out of 15 hub genes. In detail, CD79A, CXCL12, HLF and PTGDS were positively correlated with IRF4, while LAMC2, FSTL3, MT2A and TNFRSF12A were negatively related to IRF4 (Fig. 4A). In order to verify the differential expression of 16 hub genes mRNA, we analyzed the data from the TCGA database and validated that IRF4, CD79A, PDGFA, FSTL3, LAMC2, PLAUR, SEMA3C, TNFRSF12A and MT2A were upregulated in OSCC, while CFTR, CYP4F12, FUT6, HCG22, HLF, and PTGDS were downregulated in OSCC compared with Normal samples (*P* < 0.01) (Fig. 4B). For the sake of further validating differential expression of hub genes in OSCC and Normal tissues, we extracted total RNA from cell lines (HOK cell line as normal control and HN4 cell line as cancer cell) and fresh tissues (Normal tissues from healthy people and OSCC tissues from OSCC patients) respectively. QRT-PCR of HOK and HN4 showed that the expression of IRF4 in HN4 was almost twice that in HOK (Fig. 4C). QRT-PCR of Normal oral mucosa and OSCC fresh tissues presented a similar result (Fig. 4D). These results were consistent with the previous analysis results, which reminded us that IRF4 might play a positive role in transformation of OSF into OSCC.

### Identification of IRF4 as the key immune-related biomarker for transformation of OSF into OSCC through pan cancer analysis and immune infiltration analysis

**Figure 5:**
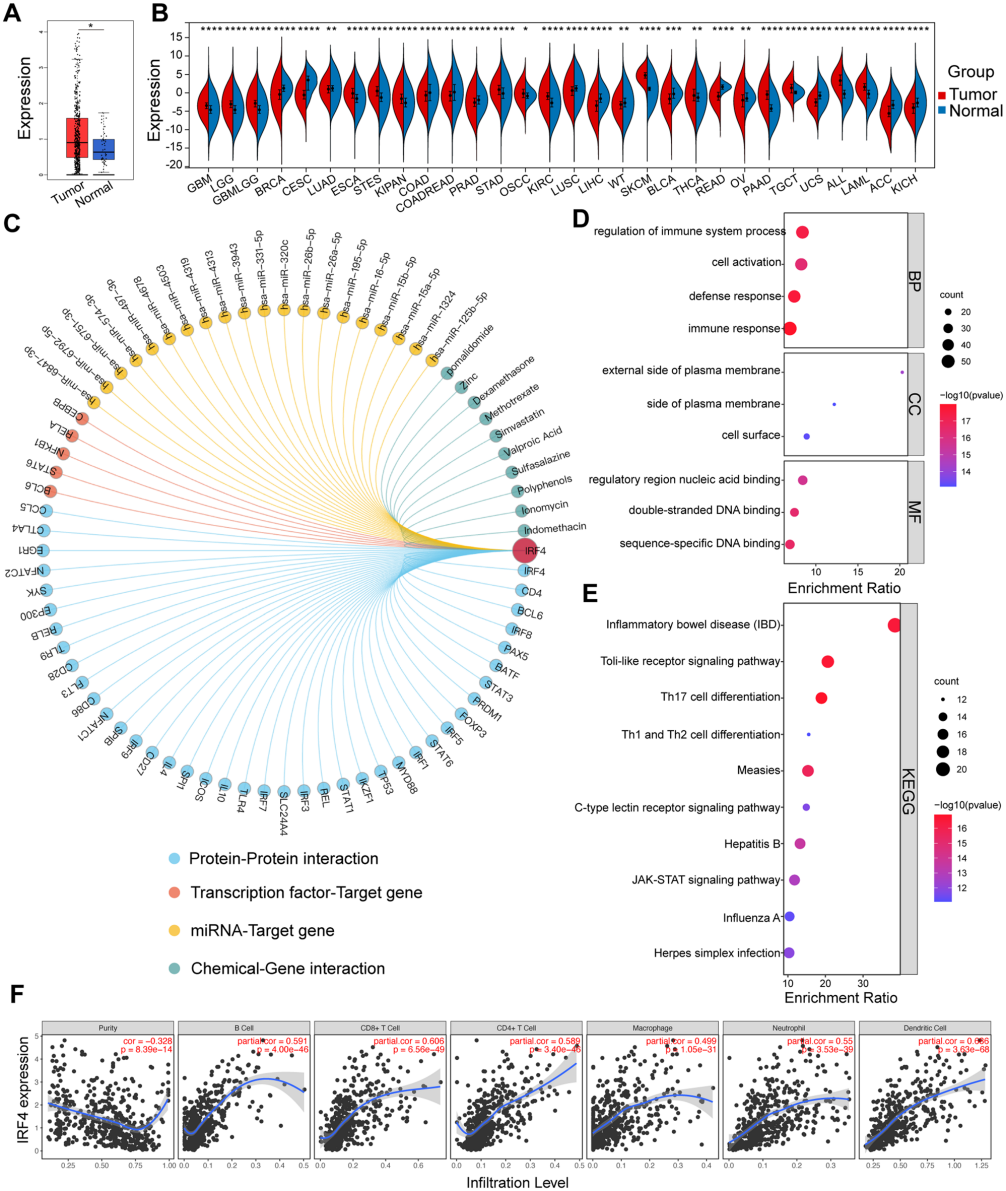
Pan Cancer Analysis and Functional Analysis of IRF4 in OSCC. (**A**) The mRNA expression of IRF4 in OSCC from TCGA database. (**B**) The expression status of IRF4 in different cancers. (**C**) The chord plot showing highly relevant molecules assigned to IRF4. (**D**) GO enrichment analysis of genes interacting with IRF4. (**E**) KEGG pathways analysis of genes interacting with IRF4. (**F**) The correlation of IRF4 and tumor infiltrating immune cells performed via TIMER. *p < 0.05; **p < 0.01; ***p < 0.001; ****p < 0.0001.

We further analyzed the expression of IRF4 mRNA in OSCC and Normal tissues using TCGA data sets in GEPIA2, and the data showed higher expression of IRF4 mRNA in OSCC than Normal mucosa (Fig. 5A), which was consistent with previous study. Pan cancer analysis revealed the difference of IRF4 in different Tumor tissues and Normal tissues (Fig. 5B). Compared with Normal tissues, the expression level of IRF4 significantly increased in GBM (glioblastoma multiforme), GBMLGG (glioma), LGG (brain lower grade glioma), ESCA (esophageal carcinoma), STES (stomach and esophageal carcinoma), KIPAN (Pan-kidney cohort), STAD (stomach adenocarcinoma), HNSC (head and neck squamous cell carcinoma), KIRC (kidney renal clear cell carcinoma), SKCM (skin cutaneous melanoma), THCA (thyroid carcinoma), PAAD (pancreatic adenocarcinoma), TGCT (testicular germ cell tumors), ALL (acute lymphoblastic leukemia) and LAML (acute myeloid leukemia), while it was significantly decreased in BRCA (breast invasive carcinoma), CESC (cervical squamous cell carcinoma and endocervical adenocarcinoma), LUAD (lung adenocarcinoma), COAD (colon adenocarcinoma), COADREAD (colon adenocarcinoma/rectum adenocarcinoma), PRAD (prostate adenocarcinoma), LUSC (lung squamous cell carcinoma), LIHC (liver hepatocellular carcinoma), WT (Wilms tumor), BLCA (bladder urothelial carcinoma), READ (rectum adenocarcinoma), OV (ovarian serous cystadenocarcinoma), UCS (uterine carcinosarcoma), ACC (adrenocortical carcinoma) and KICH (kidney chromophobe).

In order to explore possible mechanism of IRF4 in transformation of OSF into OSCC, we constructed IRF4 gene regulatory network including Protein–Protein interaction, Transcription factor-Target gene, miRNA-Target gene and Chemical-Gene interaction with Gendoma (Fig. 5C). GO functional annotation analysis of the proteins interaction with IRF4 indicated that IRF4 related genes mainly regulates immune-related functions including regulation of immune system process, defense response and immune response (Fig. 5D). KEGG pathway analysis suggested that IRF4 related genes were mainly associated with immune cell-related signaling pathway, such as inflammatory bowel disease, Th17 cell differentiation, Th1 and Th2 cell differentiation (Fig. 5E). Meanwhile, GSEA analysis of IRF4 in OSCC also showed a significant correlation immune-related GO terms and KEGG terms (Fig. S4).

**Figure 6:**
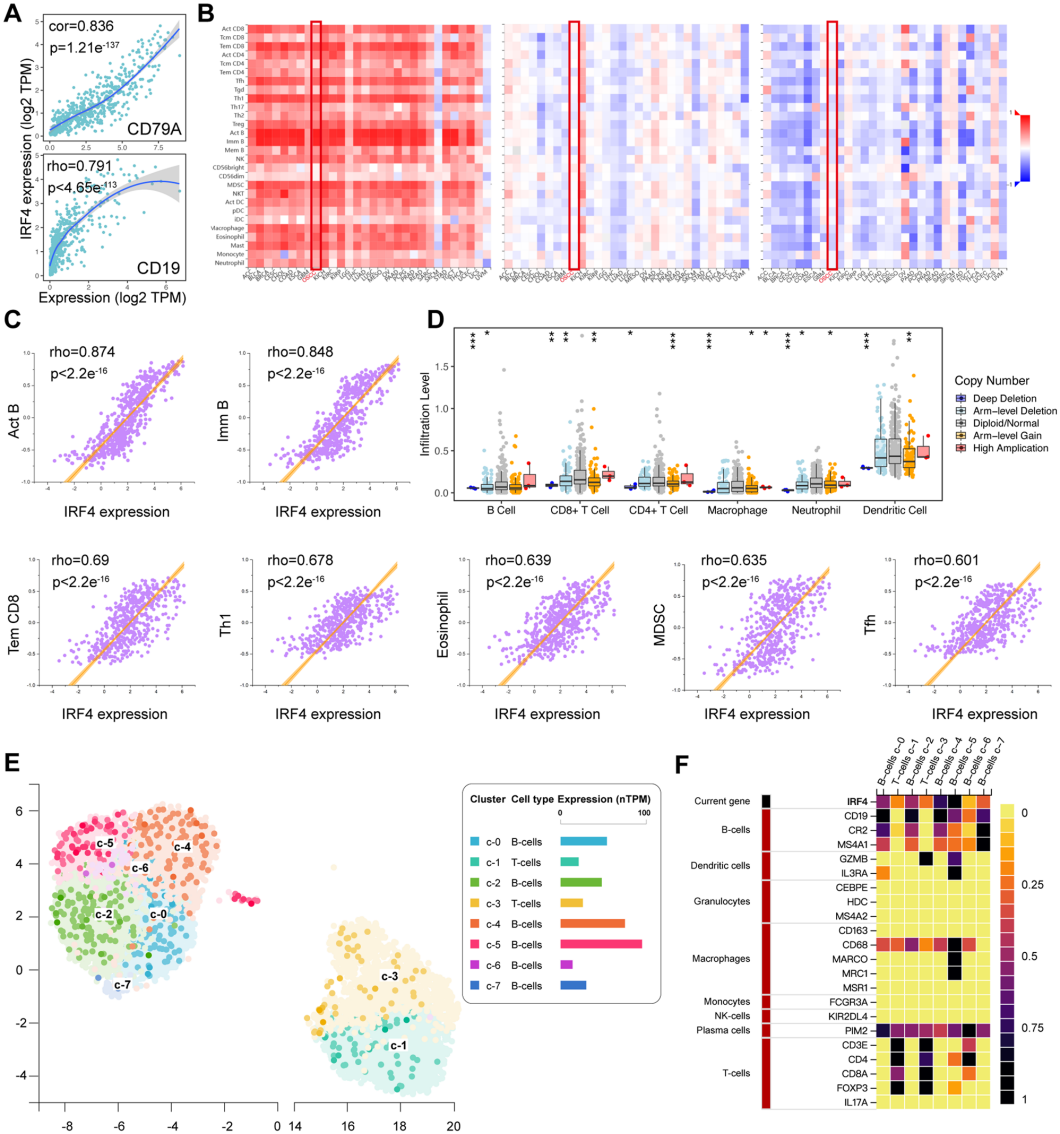
Immune Infiltration Analysis of IRF4 in OSCC. (**A**) The correlation between IRF4 and representative immune genes performed via TIMER. (**B**) The relationship between expression, copy number and methylation of IRF4 and immune infiltration levels in pan-cancer from TISIDB. (**C**) The correlation between IRF4 and multiple immune cells (Pearman’s correlation test). (**D**) The relevance of different somatic copy number alterations for IRF4 and OSCC infiltration levels from TIMER database. (**E**) Expression pattern of IRF4 Single-cell RNA in various immune cell types in lymph node from the human protein atlas. (**F**) The correlation between IRF4 and immune cell type markers from the human protein atlas.

To explore the potential immunomodulatory mechanism of IRF4 in the transformation of OSF into OSCC, we used TIMER database to evaluate the correlation between IRF4 expression in OSCC samples and immune infiltrating cells. TIMER data displayed high IRF4 expression was significantly associated with six immune cells (B cells, CD4+ T cells, CD8+ T cells, macrophages, neutrophils, and dendritic cells) in OSCC (Fig. 5F). Tumor purity is an important factor affecting the immune infiltration analysis of clinical tumor samples by genomic methods^37,38^, TIMER database also indicated that IRF4 expression levels had a significant negative correlation with tumor purity (Fig. 5F). In particular, IRF4 exhibited significantly positive correlation with the two key immune checkpoints CD79A and CD19 (Fig. 6A).

Besides, the relationship between abundance of tumor-infiltrating lymphocytes (TILs) and expression, copy number and methylation of IRF4 in pan-cancer was analyzed through TISIDB. The expression of IRF4 was positively correlated with majority of TIL levels, and the copy number and methylation of IRF4 was negatively related to immune infiltration in OSCC (Fig. 6B). In addition, rho values of spearman correlation test of IRF4 expression and abundance of Activated B cell, Immature B cell, Effector memory CD8 T cell, Type 1 T helper cell, Eosinophil, Myeloid derived suppressor cell and T follicular helper cell were greater than 0.6 (Fig. 6C). At the same time, we found that IRF4 CNV had a closely association with the degree of infiltration of B cell, CD8+ T cell, CD4+ T cell, macrophages, neutrophils and dendritic cell (Fig. 6D). Next, we used the human protein atlas to study the expression pattern of IRF4 Single-cell RNA in immune cell types, and verified the significant correlation between IRF4 and some immune cell types, such as B cells, Dendritic cells, Macrophages, Plasma cells and T cells (Fig. 6E,F).

### Validation of IRF4 at translational level

**Figure 7:**
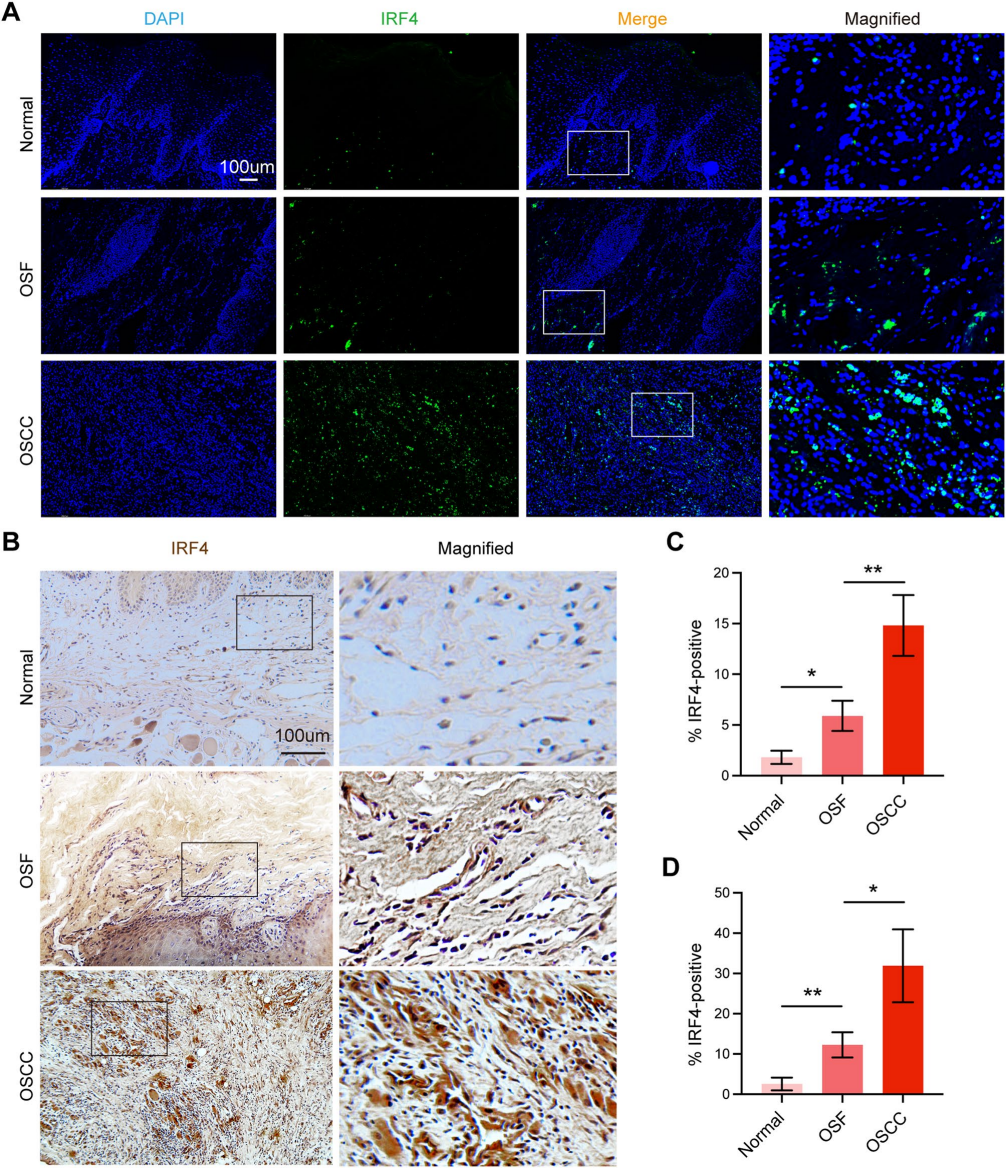
Validation of IRF4 at translational level. (**A**) IF staining of IRF4 in Normal mucosa, OSF and OSCC tissues. (**B**) IHC staining of IRF4 in Normal mucosa, OSF and OSCC tissues. (**C**) The quantitative analysis of IF staining. (**D**) The quantitative analysis of IHC staining. *p < 0.05; **p < 0.01.

Previous studies have shown a strong correlation between mRNA expression and protein expression, and mRNA upstream regulates protein translation^39–41^. There is a need for consensus on sampling, staining and quantification procedures concerning detection of IRF4 protein expression by IF and IHC staining of tissues from Normal, OSF and OSCC patients to improve reproducibility of studies and confirm bioinformatics results. Here, we detected IRF4 protein expression in different tissues from different patients by IF staining (Fig. 7A,C) and IHC staining (Fig. 7B,D), which showed a consistent, sequential upregulation of IRF4 from Normal buccal mucosa to OSF to OSCC. The results were aligned with previous studies and demonstrated that IRF4 might act as oncogene in transformation of Normal into OSF, Normal into OSCC and OSF into OSCC.

## Discussion

OSCC in the background of OSF caused by areca nut chewing has a high morbidity in some provinces of China. The five-year survival rate of OSCC has remained low in the past few decades due to the lack of effective diagnostic biomarkers^42^. Previous work has identified numerous biomarkers differentially expressed between Normal oral mucosa and OSF or OSCC tissues^43,44^. In the study, we first focused on the expression profiles and regulatory functions of IRF4 in OSCC with OSF. We identified that IRF4 was sequentially upregulated at both mRNA and protein levels from Normal oral mucosa to OSF to OSCC. In addition, we identified of IRF4 as a key immune-related biomarker and explored potential molecular mechanisms of IRF4 in transformation of OSF into OSCC.

In this study, we identified 170 (51 down- and 119 up-regulated) DEGs in total. GO analysis revealed that the DEGs is mainly associated with regulation of tube development, vasculature development, cardiovascular system development, blood vessel development, cell surface, plasma membrane region, stress fiber, growth factor activity, muscle structural constituent, and heparin binding. In addition, KEGG pathways analysis showed that DEGs mainly enriched in adrenergic signaling in cardiomyocytes, drug metabolism, metabolism of xenobiotics by cytochrome P450, chemical carcinogenesis. 170 genes were included in the PPI network and IRF4 was recognized as a potential biomarker by PCR assay and as a hub gene by bioinformatics analysis, which indicated that IRF4 may play a vital role in OSCC carcinogenesis.

The contribution of recruited immune cells to solid tumors is now a widely accepted mechanism of cancer pathogenesis^45^. Recent studies have identified the established roles of both the innate and adaptive immune systems in the host defense against highly aggressive cancers, which are driving a remarkable progresses of modern cancer immunotherapies^46^. The key role of T cells in tumor immunity has been demonstrated by the positive correlation between T-cell infiltration at the tumor bed and prognosis, and the successful clinical application of chimeric antigen receptor (CAR) T-cell infusions in treating some hematologic malignancies^47^. B cells and Tfh cells can infiltrate into tumors and B cells affect tumor progression through interaction with Tfh cells. B cells can suppress tumor progression through multiple pathways, including promoting T cell response, secreting immunoglobulins, killing cancer cells directly, and increasing tumor activity through immunosuppressive cytokines^48^. The lymphoid transcription factor, Interferon regulatory factor 4 (IRF4), is also a key regulator in the development of various immune cells, including T and B cells, suggesting its potential significant role in immuno-oncology. It is also well- known that IRF4 controls numerous decision-making processes relating to B cell differentiation, maturation, and signal transduction. Consequently, genetic alterations that affect the functional aspects of IRF4 can result in clonal transformation and have been identified in various lymphoid malignancies^49,50^. In the field of OSCC, IRF4 were highly expressed in OSCC tumor samples compared to control samples and involved in tumor immunity-related signaling pathways. IRF4 indicated significant prognostic values and could be potential therapeutic biomarkers in targeting tumor immunity of OSCC^51^.

We found IRF4 may play a role in the transformation of fibrosis into cancer. It may provide a new vision on therapeutic targets in OSCC by exploring the underlying mechanisms. Finally, it may be beneficial to realize early-stage diagnosis and provide new therapeutic targets in OSCC.

Though the study may have clinical significance, there are still several limitations we should consider. First of all, the sample numbers of both the TCGA database and clinical specimens are far from inadequate. We need to collect more information to continue verifying its accuracy. Second, the function and mechanism of biomarkers in OSF translating into OSCC need to be further studied in vivo and in vitro. In future research, we will try to explore the related molecular mechanism of IRF4 in malignant transformation of OSF into OSCC.

## Conclusion

In summary, our study identifies that IRF4 might be employed as a diagnostic and immunological predictor of malignant transformation of OSF into OSCC and provides a new vision for OSCC immunotherapy. Meanwhile, we investigated IRF4 from the aspects of methylation level, mutation analysis, qRT-PCR assay, IF and IHC analysis, which may provide ideas for future research on the role of IRF4 in the progression of OSCC.

## Supplementary Information

Supplementary Information.

## Data Availability

The datasets analyzed during the current study are available in GEO database (GSE64216, GSE23558, http://www.ncbi.nlm.nih.gov/geo) and TCGA (https://genome-cancer.ucsc.edu/).
