# Supplementary material for: IRF4 as a novel target involved in malignant transformation of oral submucous fibrosis into oral squamous cell carcinoma
Source: Sci Rep. 2023 Feb 16;13:2775. doi: 10.1038/s41598-023-29936-8 (PMC9935854; doi:10.1038/s41598-023-29936-8)
Supplement: Supplementary file 1 — Supplementary Information. [file 41598_2023_29936_MOESM1_ESM.docx]

***Supplementary Material***

**IRF4 as a novel target involved in malignant transformation of Oral Submucous Fibrosis into Oral Squamous Cell Carcinoma.**

Meng Li^1#^, Jiang Yucheng^1#^, You Jiawen^1#^, Zhao Panpan^1^, Liu Weiguang^2^, Zhao Na^1^, Zhichun Yu^3^, Ma Junqing^1,4^*

^1^Jiangsu Key Laboratory of Oral Diseases, Nanjing Medical University, 140 Hanzhong Road, Nanjing 210029, China.

^2^Department of Biochemistry, School of Life Sciences, Nanjing Normal University, Nanjing 210023, China.

^3^Green Hope High School, Cary, NC 27519, USA.

^4^Department of Orthodontics, Affiliated Hospital of Stomatology, Nanjing Medical University, Nanjing 210029, China.

**^#^**These authors contributed equally to this work.

***Correspondence**Corresponding Author: Ma Junqing, E-mail: jma@njmu.edu.cn, Fax: 0086-25-85031976.

**Keywords:** Oral squamous cell carcinoma, Oral submucous fibrosis, Biomarker, Immune infiltration, Immunotherapy.


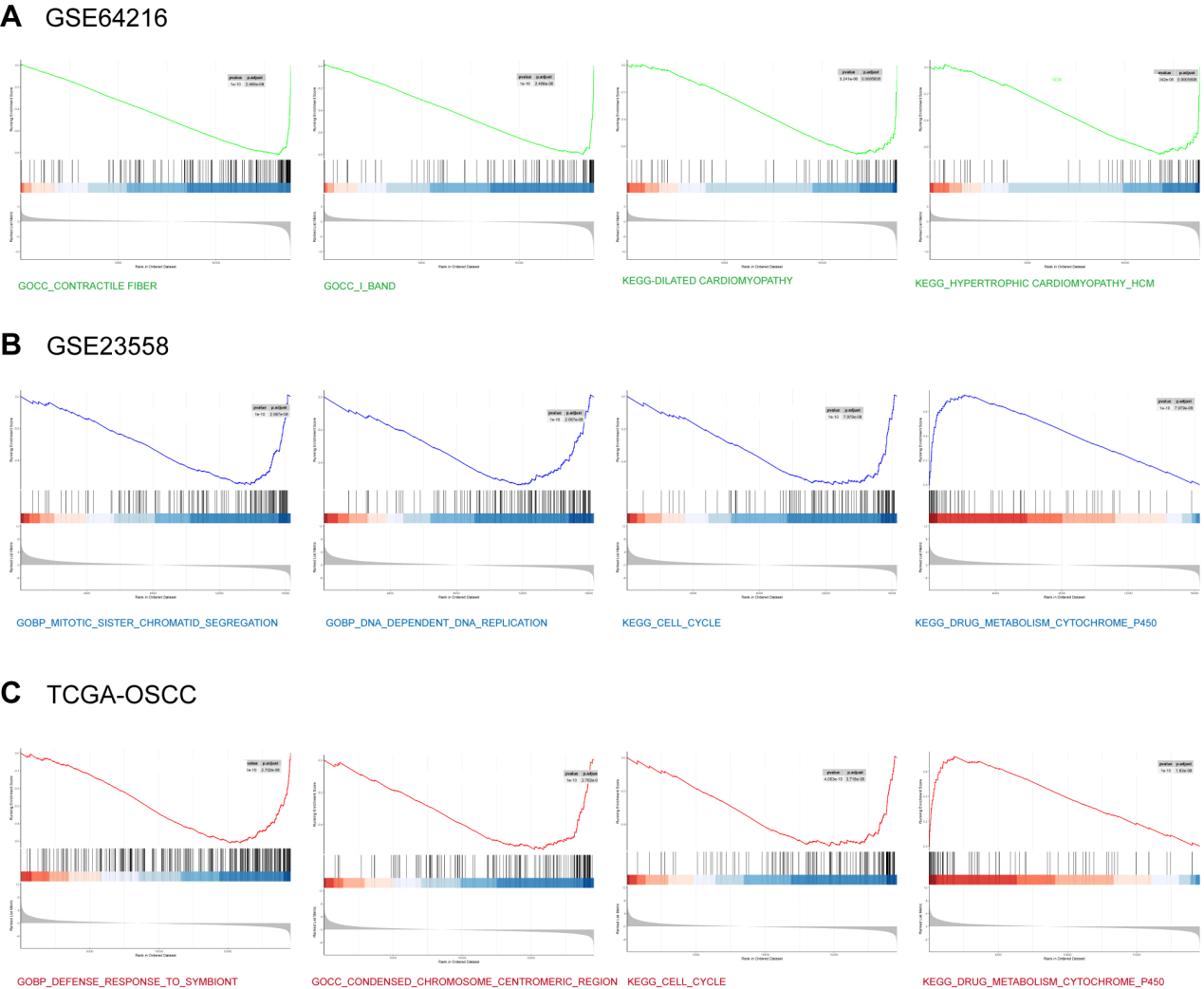


**Supplementary Figure 1.** **(A)** GO and KEGG Pathway analysis of OSF in GSE64216 using Gene set enrichment analysis (GSEA) software (http://software.broadinstitute.org/gsea/index.jsp). **(B)** GO and KEGG Pathway analysis of OSCC in GSE23558 using GSEA. **(C)** GO and KEGG Pathway analysis of OSCC in TCGA database using GSEA.


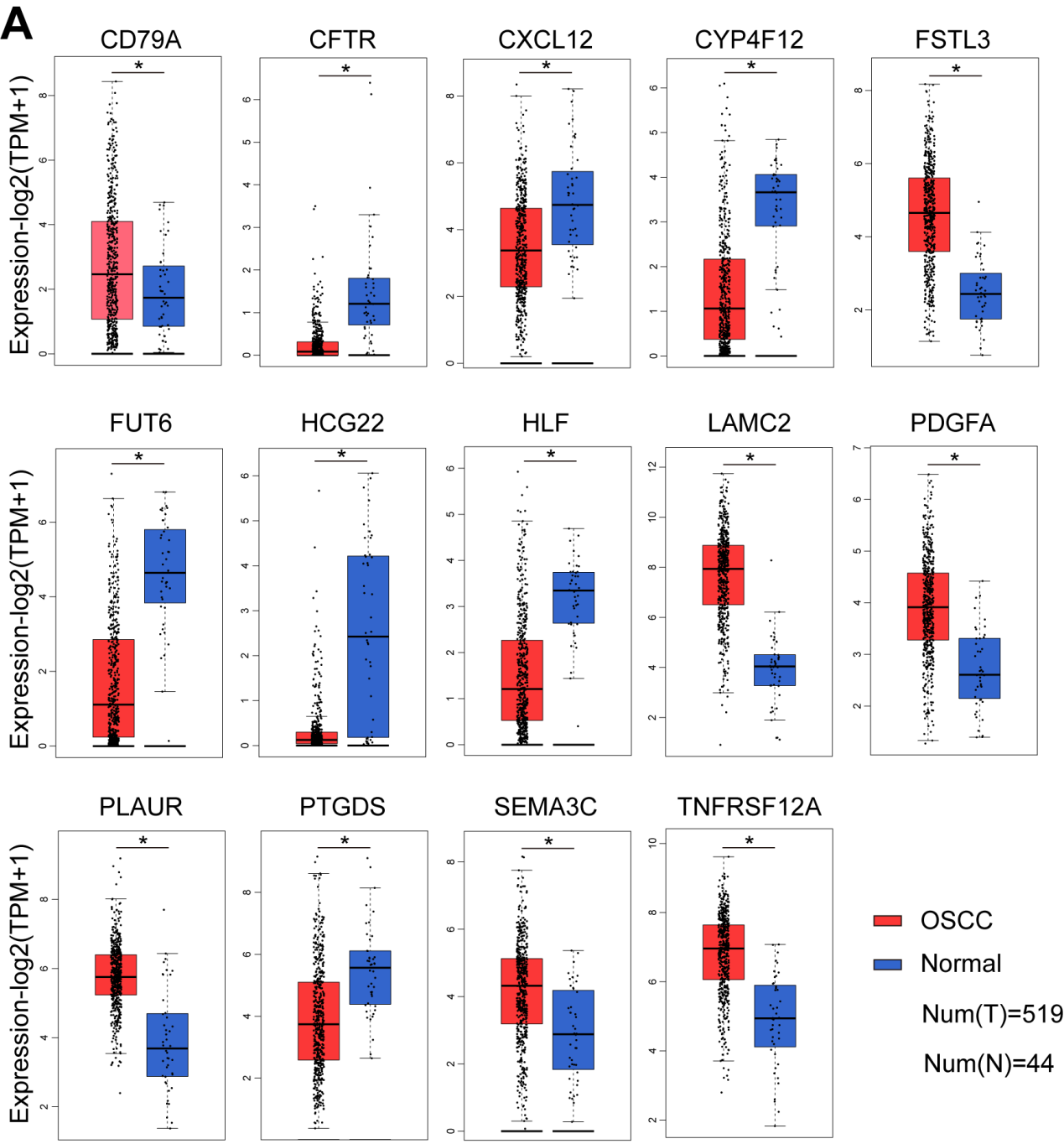
Supplementary Figure 2. (A) The mRNA expressions of DEGs (CD79A, CFTR, CXCL12, CYP4F12, FSTL3, FUT6, HCG22, HLF, LAMC2, PDGFA, PLAUR, PTGDS, SEMA3C and TNFRSF12A) between Normal and OSCC tissues from TCGA database in GEPIA2. *P < 0.05.


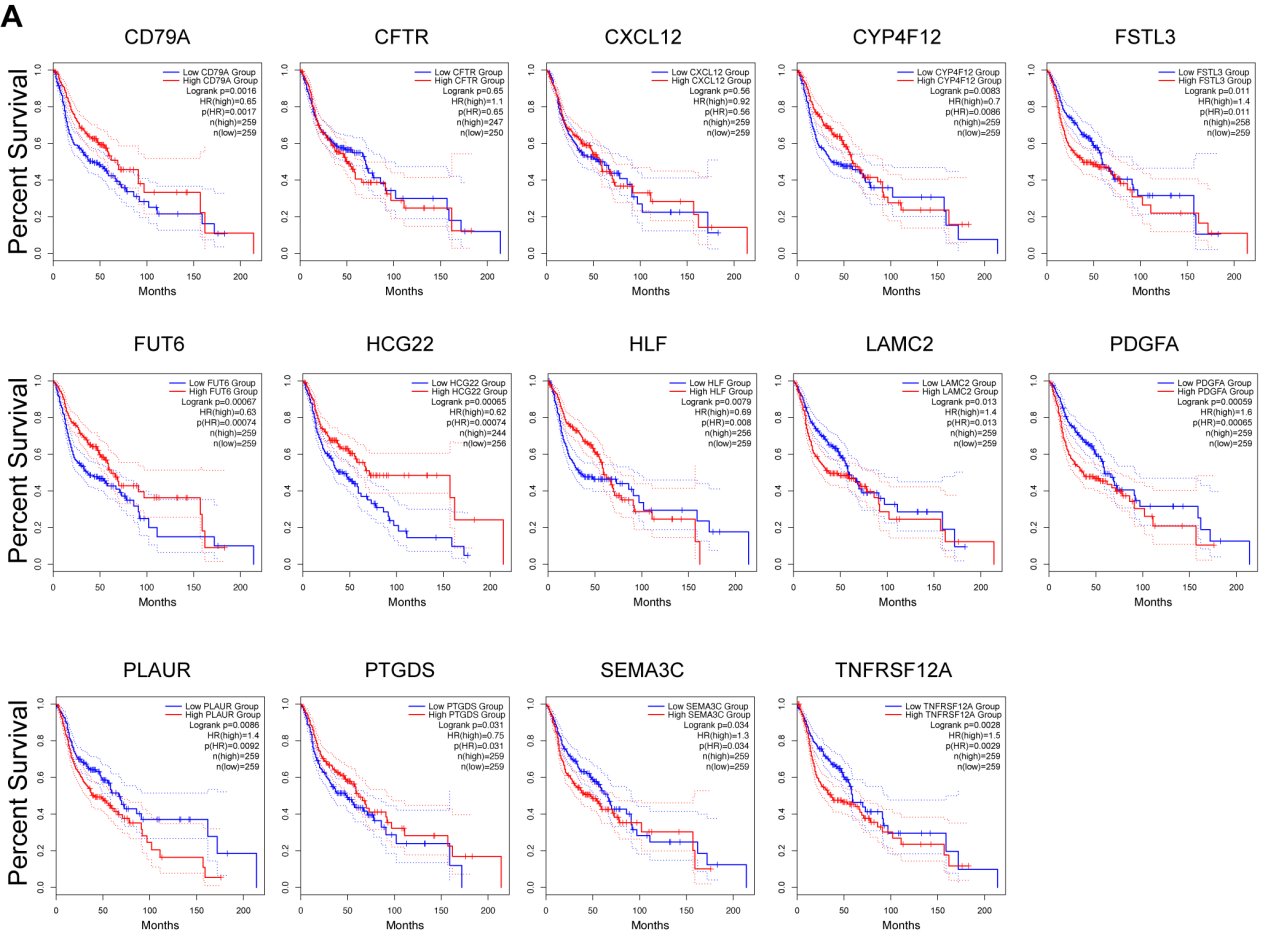
**Supplementary Figure 3. (A)** Kaplan-Meier survival curves of the association between DEGs expression and OSCC.


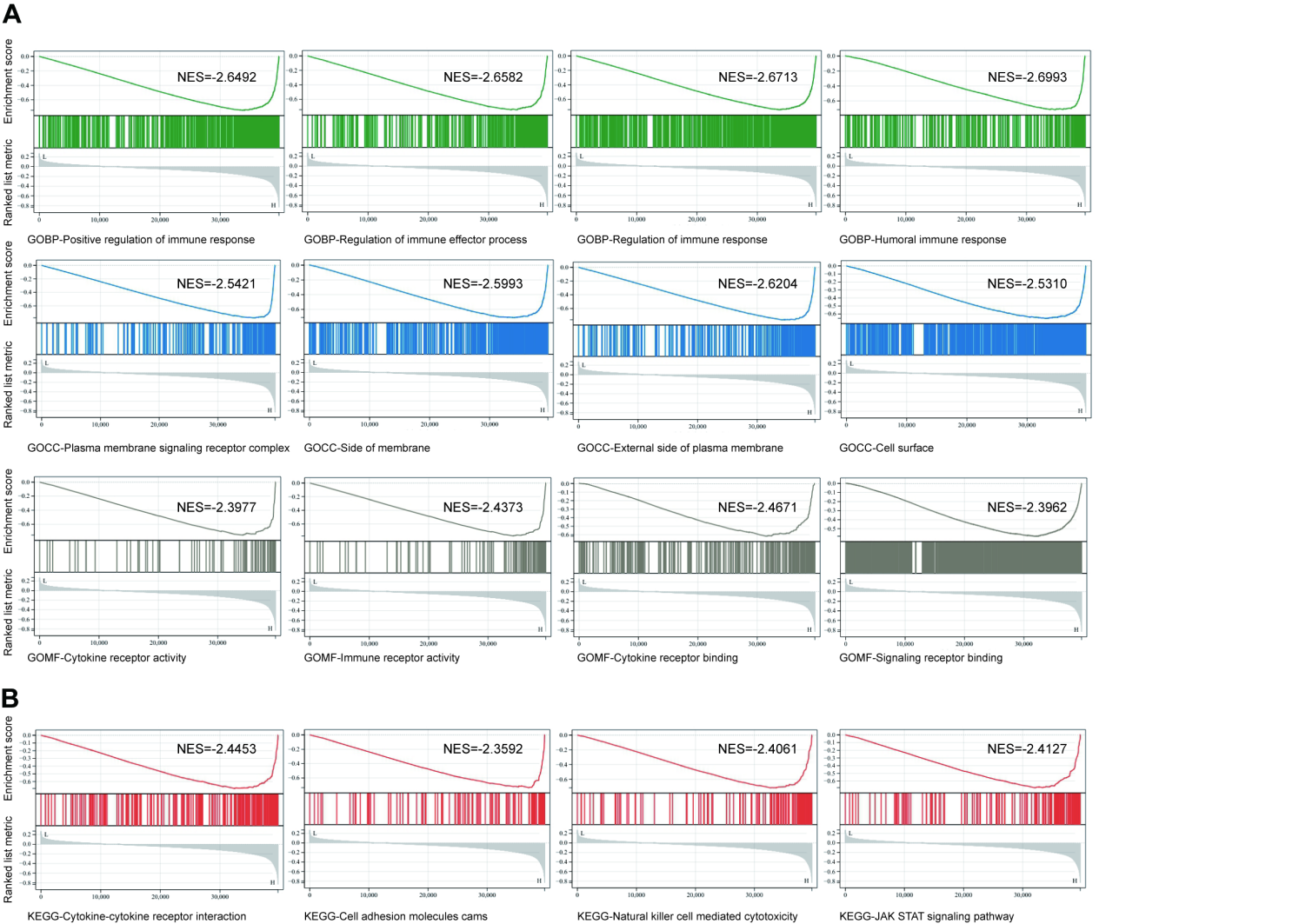
Supplementary Figure 4. (A) Gene ontology (GO) analysis of IRF4. Green indicates biological process (CC), blue indicates cellular component (CC), and grey indicates molecular function (MF). (B) KEGG analysis of IRF4 expression involved in the process of different pathways.
